# Supplementary material for: A Secreted Form of the Hepatitis E Virus ORF2 Protein: Design Strategy, Antigenicity and Immunogenicity
Source: Viruses. 2022 Sep 26;14(10):2122. doi: 10.3390/v14102122 (PMC9610824; doi:10.3390/v14102122)
Supplement: Supplementary file 1 [file viruses-14-02122-s001.zip › viruses-1892276-Supplementary.pdf]

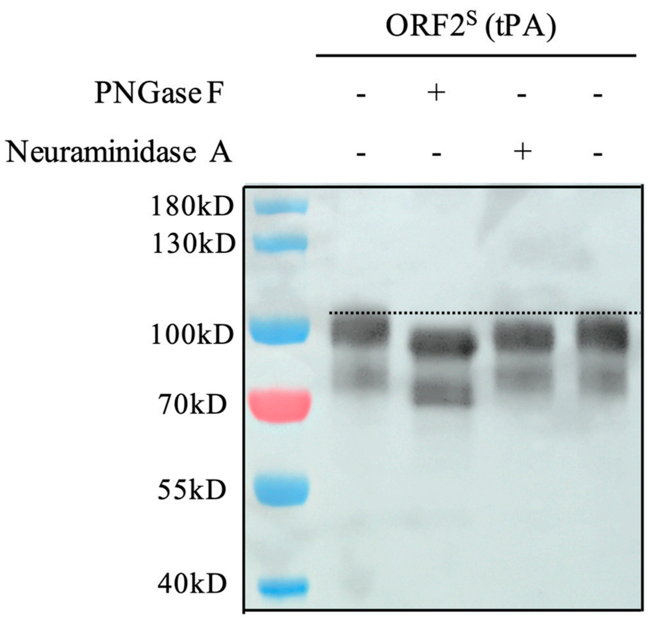

Figure S1. Glycosylation analysis of ORF2^S (tPA) protein. HEV ORF2^S (tPA) proteins were denatured and digested with indicated glycosidases (+) or not (−). The dashed line shows the mobility shift on nitrocellulose filter membrane of ORF2^S (tPA) proteins to assess the extent of de-glycosylation.

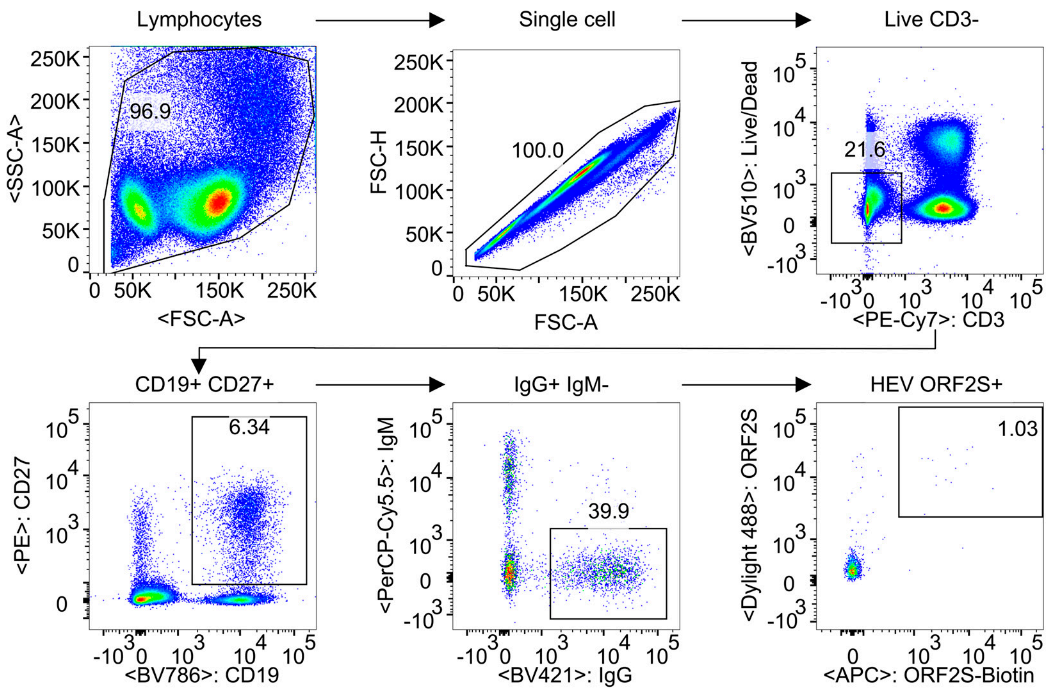

Figure S2. Flow cytometry analysis of cell. Gating strategy for recognizing ORF2^{S}-specific memory B cells through BD Arial III flow cytometer. FSC-A, forward scatter area; SSC-A, side scatter area.
